# Potential Plasma Metabolic Biomarkers of Tourette Syndrome Discovery Based on Integrated Non-Targeted and Targeted Metabolomics Screening

**DOI:** 10.1155/2022/5080282

**Published:** 2022-11-30

**Authors:** Leying Xi, Fuqiong Zhou, Wenxiu Ji, Weina Zhu, Jie Ruan, Yajie Zhang, Xueling Hu, Hongyan Long

**Affiliations:** ^1^Department of Pediatrics, Nanjing Hospital of Chinese Medicine Affiliated to Nanjing University of Chinese Medicine, Nanjing, Jiangsu, China; ^2^Department of Pediatrics, Nanjing University of Chinese Medicine, Nanjing, Jiangsu, China; ^3^Central Laboratory, Nanjing Hospital of Chinese Medicine Affiliated to Nanjing University of Chinese Medicine, Nanjing, Jiangsu, China; ^4^Clinical Biobank of Nanjing Hospital of Chinese Medicine, Nanjing Hospital of Chinese Medicine, Nanjing University of Chinese Medicine, Nanjing, Jiangsu, China; ^5^Clinical Laboratory, Nanjing Hospital of Chinese Medicine Affiliated to Nanjing University of Chinese Medicine, Nanjing, Jiangsu, China

## Abstract

**Objective:**

Tourette syndrome (TS) is a chronic neuropsychiatric disorder characterized by abnormal movements, phonations, and tics, but an accurate TS diagnosis remains challenging and indeed depends on its description of clinical symptoms. Our study was conducted to discover and verify some metabolite biomarkers based on nontargeted and targeted metabolomics.

**Methods:**

We conducted untargeted ultrahigh-performance liquid chromatography-quadrupole time-of-flight mass spectrometry (UHPLC-Q-TOF/MS) for preliminary screening of potential biomarkers on 30 TS patients and 10 healthy controls and then performed validation experiments based on targeted ultrahigh-performance liquid chromatography triple quadrupole-MS (UHPLC/MS/MS) on 35 TS patients and 14 healthy controls.

**Results:**

1775 differentially expressed metabolites were identified by partial least squares discriminant analysis (PLS-DA), fold-change analysis, *T*-test, and hierarchical clustering analysis (adjusted *p* value <0.05 and |logFC| > 1). TS plasma samples were found to be differentiated from healthy samples in our approach. Furthermore, aspartate and asparagine metabolism pathways were considered to be a significant enrichment pathway in TS progression based on metabolite pathway enrichment analysis. For the 8 metabolites involved in this pathway that we detected, we then performed validation experiments based on targeted UHPLC/MS/MS. The *t*-test, Mann–Whitney *U* test, and receiver operating characteristic (ROC) curve analysis were used to determine potential biomarkers. Ultimately, L-arginine and L-pipecolic acid were validated as significantly differentiated metabolites (*p* < 0.05), with an AUC of 70.0% and 80.3%, respectively.

**Conclusion:**

L-pipecolic acid was defined as a potential biomarker for TS diagnosis by the combined application of nontargeted and targeted metabolomic analysis.

## 1. Introduction

Tourette syndrome (TS) is a childhood-onset chronic neuropsychiatric disorder characterized by chronic muscle movements and vocal tics, lasting more than one year [1]. Some children may have attention-deficit/hyperactivity disorder (ADHD), anxiety, obsessive-compulsive disorder (OCD), and other comorbid behavioral syndromes [2], most children with TS can be expected to develop at least one comorbid disorder throughout their life, and more than half will develop two. Compared to tics, these comorbid conditions usually cause more impairment in patients with TS [3, 4]. Tics in TS typically start at 4–6 years with motor movements, such as blinking, noise twitching, and grimacing, and reach their worst severity around 10–12 years [5, 6]. Tics symptoms appear age-dependently, showing a wax and wane course, and gradually ease by late teens [5, 7]. 14 studies in mainstream schools and school-age youngsters in the community reported prevalence figures for TS between the ages of 5 and 18 years varying from 0.4% to 3.8%, and 3989 (0.949%) of 420312 young people were diagnosed as having TS; therefore, it was suggested that overall TS prevalence figure is 1% [8, 9]. In addition, studies have also shown that TS is more common in males than in females, and the ratio between males and females is about 3–4 : 1 [4, 8]. Although the etiology and pathogenesis of TS remain uncertain, it has been proved to be closely related to genetic factors, neurobiochemical factors, environmental factors, psychology, and other factors [10–13]. Besides, evidence has shown that the cortico-striatal-thalamo-cortex (CSTC) loop is closely related to the pathophysiology of TS [14].

To date, the accurate TS diagnosis has indeed depended on its clinical description of symptoms, and there are no laboratory tests for a positive diagnosis of TS and other tic disorders. The diagnosis of some patients remains challenging because of untypical early symptoms. The discovery of potential biomarkers that could help improve diagnosis is in high need. Usually, biomarkers are endogenous compounds and reflect underlying disease characteristics. Metabolomics can detect, identify, and quantify small molecular endogenous metabolites to describe biomarkers or characterize disease in biological samples, and it concludes untargeted global profiling and targeted quantification. To the best of our knowledge, no publications on the metabolomic analysis of TS patients have been reported. Therefore, the purpose of the study was to investigate the potential biomarkers in plasma of TS patients based on nontargeted and targeted metabolomic analysis.

## 2. Materials and Methods

### 2.1. Clinical Participants

From March 2020 to December 2020, TS patients and healthy controls were recruited from the Nanjing Hospital of Chinese Medicine, Nanjing Hospital of Chinese Medicine Affiliated to the Nanjing University of Chinese Medicine. TS patients were diagnosed by the Diagnostic and Statistical Manual of Mental Disorders (DSM-5®) [15]. Healthy controls without TS and other known infections were included. The study protocol conformed to the ethical guidelines of the current Declaration of Helsinki and received approval from the Ethics Committee of the Nanjing Hospital of CM. Written consent was obtained from all participants and their parents/guardians. Finally, a total of 89 participants were recruited: 30 TS samples and 10 healthy controls for untargeted UHPLC-Q-TOF/MS metabolomic analysis were included. The average age of TS patients was 7.9 years, and 21 males (70.0%) and 9 females (30.0%) were included. The mean age of healthy controls was 9.2 years, and 6 males (60.0%) and 4 females (40%) were included (sex *p*=0.559, age *p*=0.211). Another 35 TS samples and 14 healthy controls for targeted UHPLC/MS/MS analysis were included. The average age of TS patients was 8.086 years, and 24 males (69.57%) and 11 females (31.43%) were included. The mean age of healthy controls was 9.5 years, and 9 males (64.29%) and 5 females (35.71%) were included(sex *p*=0.773, age *p*=0.064). There was no statistical significance in both age and gender in TS and healthy controls. The clinical characteristics of TS patients and healthy controls are summarized in Table 1.

### 2.2. Chemicals and Reagents

HPLC grade methanol (Thermo, A456-4); HPLC grade acetonitrile (Merck, 1499230-935); ammonium acetate (Sigma, 70221); ammonium hydroxide (Fluka). Analytical standards including L-glutamic acid hydrochloride, D-ornithine monohydrochloride, L-ornithine monohydrochloride, D-homoproline, D-proline, L-arginine, L-pipecolic acid, and L(−)-carnitine were obtained from Shanghai Aladdin Biochemical Technology Co., Ltd. (Shanghai, China). Millipore-Q Water Purification System (Millipore, Germany) provided ultrahigh-purity water. All other chemicals and reagents were obtained from Merck Sigma-Aldrich (KGaA, Darmstadt, Germany).

### 2.3. Sample Preparation

Blood samples were collected from each participant; then, the samples were centrifuged for 15 min (3000 × rcf, 4°C) within 1 hour of collection. Each aliquot (1 mL) of the plasma samples was stored at −80°C until UHPLC-Q-TOF/MS and UHPLC/MS/MS processing. For the untargeted analysis, the plasma samples were thawed at 4°C. 400 *μ*L of methanol/acetonitrile (1 : 1, v/v) was added to 100 *μ*L of plasma. After 60 sec of the vortex, the mixture was stored at −20°C for 1 hour to remove protein and then centrifuged at 14,000 × rcf for 20 min at 4°C. Supernatants were subjected to UHPLC-Q-TOF/MS. Quality control (QC) samples: 10 *μ*L of each plasma sample was mixed and treated in the same way as plasma samples. The QC samples were inserted in every 8 samples to monitor the system stability of UHPLC-Q-TOF/MS. For the targeted analysis, each plasma sample was thawed at 4°C for 30 min and 200 *μ*L of aliquots was mixed with 200 *μ*L of methanol. Then, tubes were vortexed for 60 sec and then centrifuged for 15 min (13,000 × rcf, 4°C). Preparation of the standard solution: each 1 mg of the analytical standard was dissolved in methanol (1 mg/ml) and then diluted in methanol (10 *μ*g/ml). The supernatant was transferred to an autosampler vial and subjected to UHPLC/MS/MS analysis.

### 2.4. UHPLC-Q-TOF/MS Processing and Data Analysis

UHPLC-Q-TOF/MS analysis was performed on an Agilent 1290 Infinity LC system (Agilent Technologies, Santa Clara, California, USA) equipped with an AB SCIEX Triple TOF 5600 system (AB SCIEX, Framingham, MA, USA) in both positive and negative modes. Chromatographic separation was performed on ACQUITY HSS T3 1.8 *μ*m (2.1 × 100 mm) columns. The column temperature was kept at 25°C, and the flow rate was 0.3 mL/min. The UHPLC system consists of water with 25 mM ammonium acetate with 25 mM ammonia (solvent A) and acetonitrile (solvent B). The gradient profile used was optimized as follows: 0–1 min, 95% B; 1–14 min, linearly changed to 65% B; 14–16 min, linearly changed to 40% B; 16–18 min, 40% B; 18–18.1 min, linearly increased to 95% B; and 18.1–23 min, 95% B. The sample injection volume was 2 *μ*L.

Electrospray ionization (ESI) source conditions were set as follows: ion source gas 1 (gas 1) was 60 psi; ion source gas 2 (gas 2) was 60 psi; curtain gas (CUR) was 30 psi; the source temperature was set to 600°C; ion spray voltage floating (ISVF) was 5000 V (+) and −5000 V (−). Information-dependent acquisition (IDA) is a product ion scan mode based on artificial intelligence, which is used to detect and identify MS/MS spectra. Parameters were set as follows: the declustering potential (DP) was set to 60 V (+) and −60 V (−);collision energy was 35 ± 15 eV; exclude isotopes within 4 Da, candidate ions to monitor per cycle: 6.

Raw data were generated by using the ProteoWizard msConvert tool and processed by using XCMS online software (https://xcmsonline.scripps.edu/landing_page.php?pgcontent=mainPage), including nonlinear alignment, automatic integration, and peak extraction. After being normalized and integrated, MetaboAnalystR (3.0.3) [16] was employed for statistical analysis (including PLS-DA analysis, hierarchical cluster analysis, fold-change analysis, and *t*-test) and bioinformatics (pathway enrichment analysis) (https://www.metaboanalyst.ca). It performs in-house mapping of common compound names to a wide variety of database identifiers, including KEGG, HMDB, ChEBI, METLIN, and PubChem. Significance was analyzed using adjusted *p* value <0.05 and |logFC| > 1. For pathway enrichment analysis, two enrichment algorithms integrated mummichog and GSEA were used, and *p* < 0.05 was considered statistically significant.

### 2.5. Selection of Metabolites for Targeted Metabolomics

For the selection of biomarkers, the affected metabolic pathway containing abundant affected metabolites was the principal criterion. The preliminary identification of these metabolites was conducted by matching with self-constructed databases (the secondary spectral library of standard samples established in the same experimental system, about 2500 kinds). In addition, the similarity values for the accuracy of compound identification and the number of differentially expressed metabolites detected in each test sample were also important reference factors [17]. Then, selected metabolites were identified by standards and tandem mass spectrometry.

### 2.6. Sample Processing and Targeted UHPLC/MS/MS Analyses

The targeted validation of metabolites was carried out on an Agilent 1290 Infinity LC system (Agilent Technologies, Santa Clara, California, USA) equipped with an Agilent 6460 triple quadrupole mass spectrometer (Agilent Technologies). The electrospray ionization (ESI) source was set in a positive ion mode. Chromatographic separation was implemented on Agilent ZORBAX HILIC Plus columns (Agilent, USA) (50 × 2.1 mm, 1.8 *μ*m) at a column temperature of 30°C and a flow rate of 0.3 mL/min. Mobile phase A consisted of solvent A (methanol), and solvent B (0.1% formic acid in water). The gradient profile was performed as follows: 0–1 min, 5% B; 1–2 min, linearly increased to 90% B; 2–3 min, 90% B; 3–3.1 min, returned to 5%; and 3.1–4 min, 5% B. The sample injection volume was 5 *μ*L. The optimized MS settings were as follows: capillary voltage, 4000 V (+) and 3500 V (−); dry gas flow, 10 L·min∼(−1); and drying gas temperature, 350°C. Quantitation was performed using the multiple reaction monitoring (MRM) mode. The concentration data of individual metabolites were calculated directly by UHPLC/MS/MS analysis (nmol/g).

In UHPLC/MS/MS targeted analyses, Student's *t*-test and the Mann–Whitney *U* test were used for comparisons between healthy and TS patients. All statistical analyses were analyzed by using GraphPad Prism8 (GraphPad Software corporation, California, USA). Statistically, significance was defined by *p* < 0.05 (two-tailed), and the area under the curve (AUC) was calculated to further analyze differential metabolites.

## 3. Results

### 3.1. Nontargeted Metabolomic Analysis of Plasma Samples

**Figure 1:**
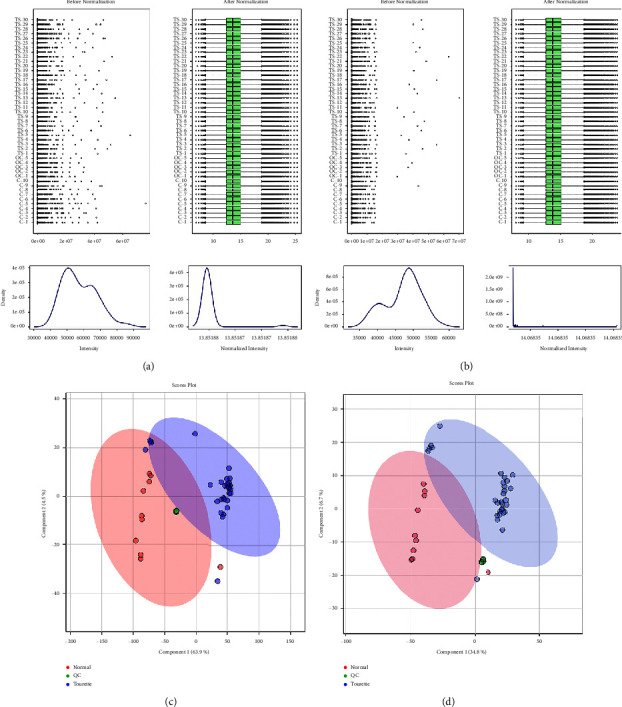
The sample distribution of TS patients and healthy controls. The distribution and intensity of input data values before (left) and after (right) normalization in the positive ion mode (a) and negative ion mode (b). PLS-DA of plasma samples of TS patients vs. healthy controls in the positive ion mode (c) and negative ion mode (d). The light red oval represents the 95% CI of the score calculated from each TS patients, and the light purple oval represents the 95% CI of the score calculated from each healthy controls.

In the nontargeted metabolic analysis of 40 plasma samples, 9003 positive-mode features and 8790 negative-mode features were extracted from each sample. Before subsequent analysis, we applied quantile normalization and log transformation to data, and the metabolomic data presented an average distribution after these processes (Figures 1(a) and 1(b)). To identify the differences of metabolite profiles between TS patients and healthy controls, the PLS-DA model was performed in both positive and negative modes, including QC samples (Figures 1(c) and 1(d)). There were significantly distinguished clusters between TS patients and healthy controls, and these results demonstrated that the metabolic profile was altered in the plasma of TS and healthy controls.

### 3.2. Identification of Differential Plasma Metabolites and Pathway Analysis

**Figure 2:**
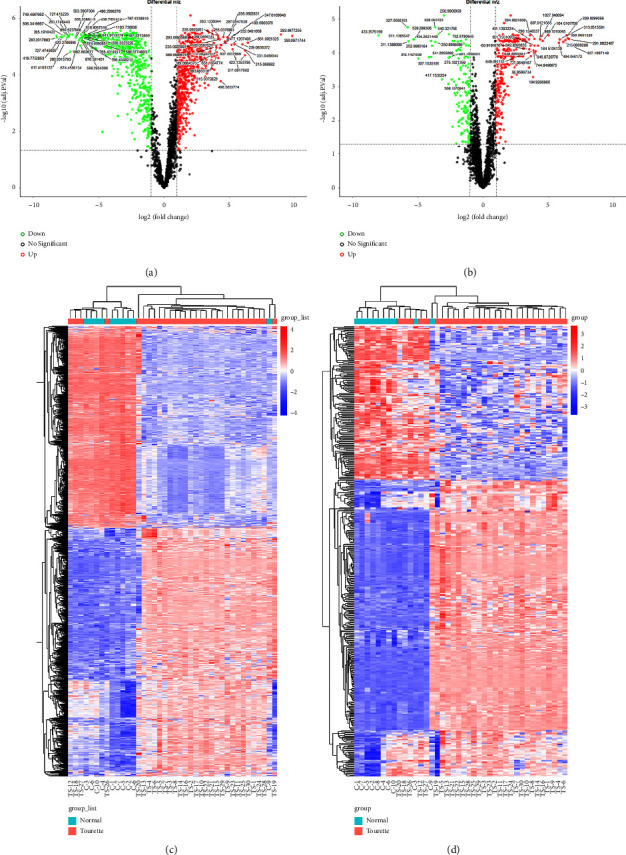
Different metabolites analysis for the data obtained from the UHPLC-Q-TOF/MS nontarget set. The volcano plot of different metabolites in the positive ion mode (a) and negative ion mode (b). The volcano plot is a combination of the fold change and *t*-test. The *x*-axis is log2 (FC), and the *y*-axis is −log10 (adj. *p* val). The red dots are logFC > 1. The green dots are logFC < −1. The heat map of clustering analysis of significant different compounds between TS patients and healthy controls in the positive ion mode (c) and negative ion mode (d).

**Figure 3:**
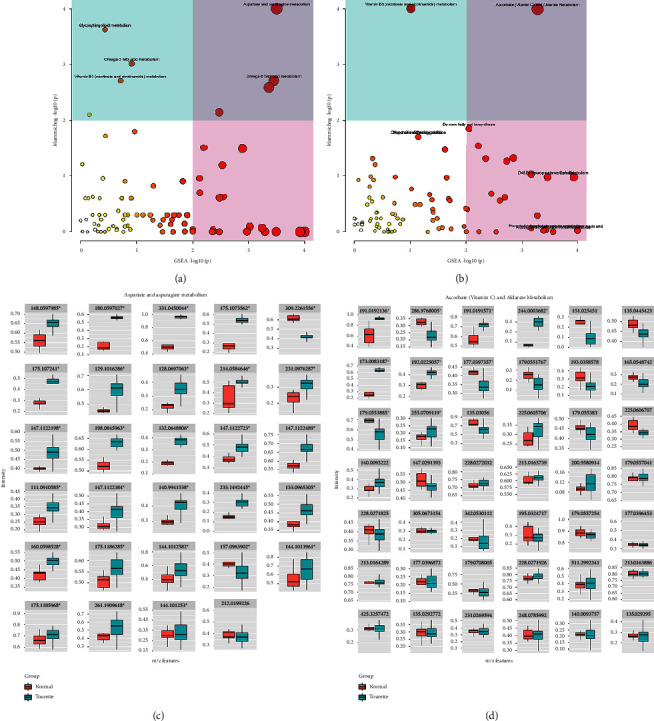
The enriched pathway analysis among significant different expression metabolites. The metabolism pathway enrichment of differential metabolites in the positive ion mode (a) and negative ion mode (b). The *x*-axis is GSEA enrichment–log10(p), and the *y*-axis is mummichog enrichment–log10(p). The matched metabolites from the different analyses in the aspartate and asparagine metabolism pathways (c) and ascorbate (vitamin C) and alternate metabolism pathways (d).

Differentiated metabolites between TS patients and healthy controls were further extracted by univariate statistical significance criteria (adjusted *p* value <0.05 and |logFC| > 1). 1324 positive-mode and 451 negative-mode different metabolite components were identified in the two groups. The volcano plot for differential metabolites between the groups is presented in Figures 2(a) and 2(b). Based on different metabolite components, the heat map of hierarchical clustering analysis was generated (Figures 2(c) and 2(d)). The satisfactory discriminatory power could be found between the groups in the heat map. Two enrichment algorithms containing mummichog and GSEA were used to analyze the enrichment pathways of differential metabolites (Figures 3(a) and 3(b)). The pathways that had the most significant combined *p* value were selected for graphic visual analysis, as shown in Figure 3(c) (positive ion mode) and Figure 3(d) (negative ion mode). There were 29 compounds matched in aspartate and asparagine metabolism pathways and 42 compounds matched in ascorbate (vitamin C) and aldarate metabolism pathways. However, compared to healthy controls, the difference in TS among these compounds had a more consistent trend in aspartate and asparagine metabolism pathways than in ascorbate (vitamin C) and aldarate metabolism pathways. Thus, we selected 29 compounds matched in aspartate and asparagine metabolism pathways for further study. Following matched the 29 compounds involved in the aspartate and asparagine metabolism pathway with the self-constructed database which has MS secondary spectrum data, we got 8 highly feasible compounds: L-Glutamate, Ornithine, D-Proline, L-Arginine, L-Pipecolic acid, L-Carnitine, D-Pipecolinic acid, and N-(omega)-Hydroxyarginine. These 8 metabolites were selected for subsequent targeted validation experiments. The basic characteristics of 8 metabolites are summarized in Table 2.

### 3.3. Validated TS Biomarkers Using Targeted Metabolomic Analysis

**Figure 4:**
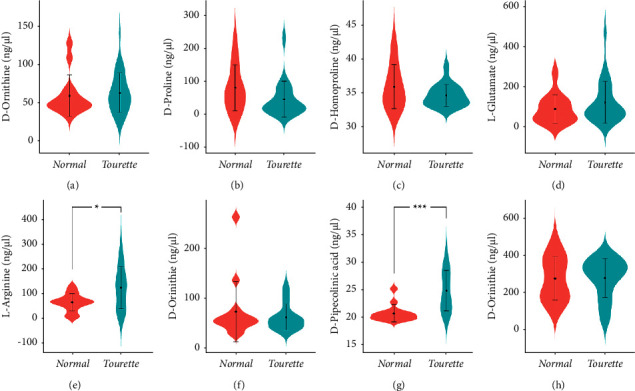
Eight candidate metabolites identified by target UHPLC/MS/MS quantitative analysis. D-ornithine (a), D-proline (b), D-homoproline (c), L-glutamate (d), L-arginine (e), L-ornithine (f), D-pipecolinic (g), and L-carnitine (h) levels between TS patients and healthy controls in plasma. The lines show interquartile ranges, and dots show medians.^*∗*^*p* < 0.05, ^*∗∗∗*^*p* < 0.001.

The specific concentration of 8 metabolites was determined by UHPLC/MS/MS (Supplementary Figure S2). Selected MRM transitions and optimized conditions for MS are summarized in Table 3. Every metabolite linear standard curve was generated from mixed standard solutions (Supplementary Figure S3). The *R*^2^ values of 8 metabolites stand curve linearity were all greater than 0.99, and these results indicate their accurate concentration calculation based on these curves (Supplementary Table S1). The concentrations of L-glutamate, D-ornithine monohydrochloride, L-arginine, L-carnitine, and D-homoproline increased in TS plasma than in healthy plasma, and the concentrations of D-proline, L-pipecolic acid, and L-ornithine monohydrochloride decreased in TS plasma. Two (L-arginine and L-pipecolic acid) of 8 verified metabolites had significant differences (*p* < 0.05) (Figure 4).

### 3.4. L-Pipecolic Acid Could Be Used as a New Biomarker for TS Diagnosis

**Figure 5:**
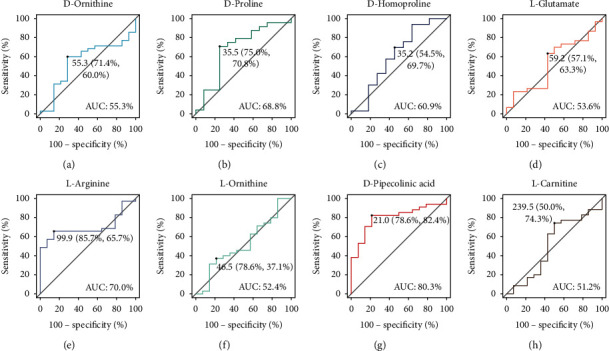
The ROC curve analysis of candidate metabolite amino acids in the UHPLC/MS/MS-targeted group. D-ornithine, AUC = 55.3% and Youden index = 55.3 (71.4%, 60.0%) (a), D-proline, AUC = 68.8% and Youden index = 35.5 (75.0%, 70.8%) (b), D-homoproline, AUC = 60.9% and Youden index = 35.2 (54.5%, 69.7%) (c), L-glutamate, AUC = 53.6% and Youden index = 59.2 (57.1%, 63.3%) (d), L-arginine, AUC = 70.0% and Youden index = 99.9 (85.7%, 65.7%) (e), L-ornithine, AUC = 52.4% and Youden index = 46.5 (78.6%, 37.1%) (f), D-pipecolinic, AUC = 80.3% and Youden index = 21.0 (78.6%, 82.4%) (g), and L-carnitine, AUC = 51.2% and Youden index = 239.5 (50.0%, 74.3%) (h). AUC (50%–70%), low accuracy; AUC (70%–90%), moderate accuracy; AUC (>90%), high accuracy.

To evaluate the diagnostic value of 8 metabolites for TS, the ROC curve analysis of these metabolites was performed, and we calculated their area under the curve (AUC) and Youden indexes. As shown in Figure 5, D-Ornithine (AUC = 55.3%),D-Proline(AUC = 68.8%),D-Homoproline(AUC = 60.9%), L-Glutamate (AUC = 53.6%),L-Arginine (AUC = 70.0%), L-Ornithine (AUC = 52.4%), D-Pipecolinic acid (AUC = 80.3%), L-Carnitine (AUC = 51.2%). L-pipecolic acid had a better diagnostic value for TS (AUC > 80%). The optimal threshold was 99.9 ng/*μ*L for L-arginine, and it was 21.0 ng/*μ*L for D-pipecolinic acid in plasma.

## 4. Discussion

Tourette syndrome is a complex neurological disorder characterized by repetitive, sudden, involuntary motor, and phonic tics. Most children with TS can be estimated to develop other associated comorbid conditions. However, there are problems with the actual diagnosis of TS. Untypical early symptoms and complicated symptoms of TS patients still pose a challenge to its diagnosis. There is a high need for discovering some biomarkers that could help improve diagnosis. Metabolomics provides us a unique perspective to understand the regulation of metabolic networks in the biological system. Furthermore, it is also emerging as a new tool to research the central nervous system, and there are already some research studies on metabolomic signatures in schizophrenia, depression, and bipolar disease [18–21]. To the best of our knowledge, this was the first study to explore potential plasma metabolic biomarkers of TS through nontargeted combined with targeted metabolic profiling.

In untargeted metabolomics analysis, there was a clear distinction between TS plasma and healthy plasma, and this also reflected our strict inclusion criteria of TS patients based on the existing clinical diagnosis. Furthermore, aspartate and asparagine metabolism pathways were found to be significantly affected by TS, and 8 differential metabolites in these pathways were identified by matching with self-constructed databases (D-ornithine, D-proline, D-homoproline, L-glutamate, L-arginine, L-ornithine, D-pipecolinic acid, and L-carnitine). To further validate the potential biomarkers in TS patients, UHPLC/MS/MS-targeted quantitative analysis was performed. At this stage, we collected TS patients as possible as we could. However, the sample size of TS patients in the study is limited because of the low incidence and informed consent of parents of children. The final results showed that only 2 (L-arginine and L-pipecolic acid) of the 8 metabolites had statistical differences between TS patients and healthy controls (*p* < 0.05). Coincidentally, L-pipecolic acid also performed better in the area under the curve analysis than other 7 metabolites (AUC = 80.3%). We also tried to perform stratified k-fold validation (*k* = 5) for ROC analyses. Honestly, stratified sampling AUC results were so unstable to determine which metabolites were better biomarkers for TS (Supplementary Table S2). We recognized some limitations in this work, among which is the small size of the cohorts, which might be the core reason why cross-validation was not stable. However, the results indicated that the AUC value of D-pipecolinic acid was always more than 0.7 in 5 batches of stratified samplings. So we thought it could serve as a biomarker with more robustness.

The etiology and pathogenesis of TS are still uncertain, but the CSTC circuit appears to be closely related to the pathophysiology of TS, and some amino acids, including GABA and glutamate, act as neurotransmitters and neuromodulators, play a key role in CSTC circuitry, and participate in habitual behavior formation and pathophysiology of tics [22–24]. As the main regulatory element in the CSTC circuit, the role of the striatum in TS was also reported in large studies [25–27]. Studies found that glutamate increased in TS, while in our study, glutamate showed no statistical differences between TS patients and healthy controls, which may be due to our limited size of samples. Besides, aspartate and asparagine metabolism pathways, L-arginine and L-pipecolic acid, found in the study also indicate that amino acids participate in the pathogenesis of TS. As precursors and intermediates of neurotransmitters, amino acids play an important role in neurotrophic development and information transmission. It is difficult to determine the amino acid level of brain tissue and cerebrospinal fluid in TS patients, while free amino acids in human body fluid could be stable in the TS active period. In addition, amino acids could pass through the blood-brain barrier, so the level of amino acids in blood can also indirectly reflect the situation of amino acids in the brain.

Aspartate and asparagine are two nonessential amino acids with similar structures. Aspartate exists in two enantiomeric forms, L-aspartic acid and D-aspartic acid. As excitatory neurotransmitters in the central nervous system, aspartate takes part in the long-range information exchange via activation of glutamate receptor channels [28], while high levels of aspartate could reduce synaptic plasticity, impair cognitive function, and early spatial memory [29, 30]. N-methyl-D-aspartic acid (NMDA) is a high-energy form of aspartic acid and one of the well-known agonists for a class of glutamate receptors. Aspartate can selectively activate extrasynaptic NR1-NR2B NMDA receptors. Aspartate participates in immature neurons through activating NR1-NR2B receptors, results in the substantial Ca^2+^ influx, then activates cAMP-dependent gene transcription, and inhibits cAMP-responseelement-binding protein (CREB) function, reducing the expression of brain-derived neurotrophic factor (BDNF) and inducing excitotoxic neuronal death in mature neurons [31]. Excessive activation of NMDA receptors is associated with memory, learning impairment, and excitotoxic cell death [32–35]. It is interesting to note that previous studies showed that the number of NMDA receptors in the hippocampus decreases with aging [34], while symptom intensity and frequency of TS also decrease with age, and whether this feature of TS is related to NMDA receptors still needs to be further studied. In addition, N-acetylaspartate (NAA), a noninvasive marker for neuronal health, which is synthesized from aspartate and acetyl coenzyme A in neurons, reflects the extent of neuronal impairment and dysfunction. Several studies have found that the decreased concentration of NAA is involved inneuropsychiatric disorders listed in DSM-IV 1R, the acknowledged compendium of clinical psychiatric diseases [36, 37]. There are a few kinds of research between NAA and TS. One study found lower levels of NAA in the left putamen and frontal cortex in TS plasma and suggested the compromised neuronal integrity and insufficient density of neuronal and nonneuronal cells [38]. Taken together, these research studies suggested that aspartate and asparagine metabolism pathways have a close relationship with the pathogenesis of TS.

In this study, the levels of L-arginine were upregulated in TS patients. L-arginine is a semiessential amino acid involved in the synthesis of L-ornithine, L-glutamate, and polyamines and a precursor for nitric oxide (NO) synthesis [39]. The L-arginine/NO pathway is involved in physiological processes, such as vasodilation, memory, neuroprotection, and immune defense in cardiovascular, immune, and nervous systems [40, 41]. The effects of L-arginine in the nervous system are often attributed to NO. In the nervous system, NO acts as a neurotransmitter and plays an important role in synaptic plastic, neural development, regeneration, transcriptional activity, learning and memory, and neuroprotection [42–44]. Small quantities of NO are neuroprotective, and an excessive amount of NO becomes noxious, which could cause cell damage and is involved in various disorders, such as major depressive disorder (MDD), autism spectrum disorder (ASD), obsessive-compulsive disorder (OCD), Alzheimer's disease (AD), attention-deficit hyperactivity disorder (ADHD), and other neurodegenerative disorders [40, 45–49]. The role of L-arginine and NO in TS has not been explored. Our results for the first time showed that the concentration of L-arginine was higher in TS patients, and whether the L-arginine/NO pathway is involved in the development of TS needs to be further studied.

Pipecolic acid (PA) is a metabolite of lysine and has two different enantiomers, including L-pipecolic acid (L-PA) and D-pipecolic acid (D-homoproline; D-PA). L-PA is a significant marker for the diagnosis of peroxisomal disorders. D-PA is believed to originate mainly from the catabolism of dietary lysine by intestinal bacteria and found to increase in patients with liver diseases [50, 51]. PA is known to be a GABA receptor agonist, which could inhibit neuronal GABA uptake and/or enhance its release, while D-PA is found to be more effective in restraining the actions of the central GABA system than L-PA acid [52, 53]. GABA is a major inhibitory neurotransmitter involved in the CSTC pathway, and its dysfunction has a close relationship with TS. In this study, D-pipecolic acid increased in TS and displayed moderate efficiency (AUC = 80.3) as a potential biomarker. We speculated that D-PA may participate in the pathophysiology of TS through the central GABA system.

However, there are two limitations in the study. First, 89 TS patients and healthy controls were recruited; however, the size of samples was limited. Second, the global generalizability of the findings may be affected because all participants recruited in this study were from China. Furthermore, a large number of participants are needed to validate the potential utility of these plasma metabolic biomarkers for the diagnosis of TS.

## 5. Conclusion

In summary, we employed UHPLC/Q-TOF/MS nontargeted analysis and UHPLC/MS/MS-targeted quantitative analysis to identify the plasma metabolic profile in TS. Aspartate and asparagine metabolism pathways were found to be significantly affected by TS. L-pipecolic acid may be used as a potential diagnostic biomarker for TS. Furthermore, our study also confirmed that the imbalance of amino acid neurotransmitters is closely associated with the pathophysiology of TS, while the role of amino acids in TS still deserves further exploration.

## Figures and Tables

**Table 1:** Clinical characteristic of TS patients and healthy controls.

	TS patients (*n* = 65)	Healthy controls (*n* = 24)
Mean age (years)	7.99	9.35
Gender (males/females)	45/20	15/9
Facial tics (%)	100	—
Limb tics (%)	93.8	—
Complex motor tics (%)	95.4	—
Complex vocal tics (%)	9.2	—
Patients with comorbid ADHD (%)	49.2	—
Patients with comorbid OCD (%)	23.1	—
Patients with comorbid depression (%)	7.7	—

**Table 2:** Differential metabolites identified by logFC and adjusted *p* value.

Metabolites	M/Z	Retention time	logFC	Adjust. *p* value
L-carnitine	162.112	710.475	1.9373	<0.0001
L-glutamate	148.0598	786.404	1.3103	<0.0001
Ornithine	133.0965	1009.645	1.1201	0.0011
D-proline	116.0699	1009.77	1.1579	0.0030
L-arginine	175.1186	1029.94	0.7928	0.0477
N-(omega)-hydroxyarginine	232.14	866.86	1.8824	0.0017
D-pipecolinic acid	130.0856	1047.88	1.0188	0.0004
L-pipecolic acid	147.1122	1047.48	1.2865	<0.0001

**Table 3:** Selected MS/MS channels and parameters.

Component	Parent ion	Daughter ion	Fragmentor (V)	Collision energy (V)
L(−)-carnitine	162.0	60.0	115	17
L-glutamic acid hydrochloride	148.0	84.0	85	13
L-ornithine monohydrochloride	133.0	70.1	70	17
D-proline	116.0	70.1	85	21
L-arginine	175.1	70.2	110	29
D-ornithine monohydrochloride	133.0	70.0	60	21
D-homoproline	130.0	84.1	75	13
L-pipecolic acid	130.0	83.9	85	17

## Data Availability

The data that support the findings of this study are available from the corresponding author upon reasonable request.

## Additional Points

*Code Availability.* Raw metabolomic data were processed using ProteoWizard and XCMS software. Metabolomic analyses were performed using R package MetaboAnalystR (3.0.3). Statistical analyses were performed using R version 4.0.3 software.

## Supplementary Materials

Supplementary Materials Supplementary Table S1: the standard curve formulas of metabolite standards. Supplementary Table S2: the AUCs of ROC through stratified 5-fold validation. Supplementary Figure S1: the workflow diagram of this experiment, including preliminary screening of differential metabolites in the nontargeted group and validation in the targeted group. Supplementary Figure S2: the quantitative detection of D-ornithine (A), D-proline (B), D-homoproline (C), L-glutamate (D), L-arginine (E), L-ornithine (F), D-pipecolinic (G), and L-carnitine (H) by ESI in the positive mode using MRM. Supplementary Figure S3: The standard curve of metabolite standards based on the UHPLC/MS/MS platform, including D-ornithine (A), D-proline (B), D-homoproline (C), L-glutamate (D), L-arginine (E), L-ornithine (F), D-pipecolinic (G), and L-carnitine (H).

## References

[1] Leckman J. F. (2002). Tourette’s syndrome. *The Lancet*.

[2] Cravedi E., Deniau E., Giannitelli M. (2018). Disentangling Tourette syndrome heterogeneity through hierarchical ascendant clustering. *Developmental Medicine and Child Neurology*.

[3] Bernard B. A., Stebbins G. T., Siegel S. (2009). Determinants of quality of life in children with Gilles de la Tourette syndrome. *Movement Disorders*.

[4] Hirschtritt M. E., Lee P. C., Pauls D. L. (2015). Lifetime prevalence, age of risk, and genetic relationships of comorbid psychiatric disorders in Tourette syndrome. *JAMA Psychiatry*.

[5] Bloch M. H., Leckman J. F. (2009). Clinical course of Tourette syndrome. *Journal of Psychosomatic Research*.

[6] Leckman J. F., Zhang H., Vitale A. (1998). Course of tic severity in Tourette syndrome: the first two decades. *Pediatrics*.

[7] Peterson B. S., Leckman J. F. (1998). The temporal dynamics of tics in Gilles de la Tourette syndrome. *Biological Psychiatry*.

[8] Robertson M. M. (2008). The prevalence and epidemiology of Gilles de la Tourette syndrome. Part 1: the epidemiological and prevalence studies. *Journal of Psychosomatic Research*.

[9] Robertson M. M. (2008). The prevalence and epidemiology of Gilles de la Tourette syndrome. Part 2: tentative explanations for differing prevalence figures in GTS, including the possible effects of psychopathology, aetiology, cultural differences, and differing phenotypes. *Journal of Psychosomatic Research*.

[10] Brander G., Rydell M., Kuja-Halkola R. (2018). Perinatal risk factors in Tourette’s and chronic tic disorders: a total population sibling comparison study. *Molecular Psychiatry*.

[11] Gagne J. P. (2019). The psychology of Tourette disorder: revisiting the past and moving toward a cognitively-oriented future. *Clinical Psychology Review*.

[12] Hongyan L., Mengjiao Z., Chunyan W., Yaruo H. (2019). Rhynchophyllin attenuates neuroinflammation in Tourette syndrome rats via JAK2/STAT3 and NF-*κ*B pathways. *Environmental Toxicology*.

[13] Qi Y., Zheng Y., Li Z., Liu Z., Xiong L. (2019). Genetic studies of tic disorders and tourette syndrome. *Methods in Molecular Biology*.

[14] Albin R. L., Mink J. W. (2006). Recent advances in Tourette syndrome research. *Trends in Neurosciences*.

[15] Association A. P. (2013). *Diagnostic and Statistical Manual of Mental Disorders (DSM-5®)*.

[16] Pang Z., Chong J., Li S., Xia J. (2020). MetaboAnalystR 3.0: toward an optimized workflow for global metabolomics. *Metabolites*.

[17] Chen Y., Ma Z., Shen X. (2018). Serum lipidomics profiling to identify biomarkers for non-small cell lung cancer. *BioMed Research International*.

[18] Kaddurah-Daouk R., Krishnan K. R. R. (2009). Metabolomics: a global biochemical approach to the study of central nervous system diseases. *Neuropsychopharmacology*.

[19] Kaddurah-Daouk R., McEvoy J., Baillie R. A. (2007). Metabolomic mapping of atypical antipsychotic effects in schizophrenia. *Molecular Psychiatry*.

[20] Lan M. J., McLoughlin G. A., Griffin J. L. (2009). Metabonomic analysis identifies molecular changes associated with the pathophysiology and drug treatment of bipolar disorder. *Molecular Psychiatry*.

[21] Paige L. A., Mitchell M. W., Krishnan K. R. R., Kaddurah-Daouk R., Steffens D. C. (2007). A preliminary metabolomic analysis of older adults with and without depression. *International Journal of Geriatric Psychiatry*.

[22] Gasbarri A., Pompili A., Packard M. G., Tomaz C. (2014). Habit learning and memory in mammals: behavioral and neural characteristics. *Neurobiology of Learning and Memory*.

[23] Mahone E. M., Puts N. A., Edden R. A. E., Ryan M., Singer H. S. (2018). GABA and glutamate in children with Tourette syndrome: a (1)H MR spectroscopy study at 7T. *Psychiatry Research: Neuroimaging*.

[24] Singer H. S. (2016). Habitual and goal-directed behaviours and Tourette syndrome. *Brain*.

[25] Caligiore D., Mannella F., Arbib M. A., Baldassarre G. (2017). Dysfunctions of the basal ganglia-cerebellar-thalamo-cortical system produce motor tics in Tourette syndrome. *PLoS Computational Biology*.

[26] Kataoka Y., Kalanithi P. S., Grantz H. (2010). Decreased number of parvalbumin and cholinergic interneurons in the striatum of individuals with Tourette syndrome. *Journal of Comparative Neurology*.

[27] Rizzo F., Nespoli E., Abaei A. (2018). Aripiprazole selectively reduces motor tics in a young animal model for tourette’s syndrome and comorbid attention deficit and hyperactivity disorder. *Frontiers in Neurology*.

[28] Toyota M., Spencer D., Sawai-Toyota S. (2018). Glutamate triggers long-distance, calcium-based plant defense signaling. *Science*.

[29] Cui B., Wu M., She X., Liu H. (2012). Impulse noise exposure in rats causes cognitive deficits and changes in hippocampal neurotransmitter signaling and tau phosphorylation. *Brain Research*.

[30] Humphries P., Pretorius E., Naude H. (2008). Direct and indirect cellular effects of aspartame on the brain. *European Journal of Clinical Nutrition*.

[31] Nadler J. V. (2011). Aspartate release and signalling in the hippocampus. *Neurochemical Research*.

[32] Gillessen T., Budd S. L., Lipton S. A. (2002). Excitatory amino acid neurotoxicity. *Advances in Experimental Medicine & Biology*.

[33] Tsoka P., Barbisan P. R., Kataoka K. (2019). NLRP3 inflammasome in NMDA-induced retinal excitotoxicity. *Experimental Eye Research*.

[34] Yamada K., Nabeshima T. (1998). Changes in NMDA receptor/nitric oxide signaling pathway in the brain with aging. *Microscopy Research and Technique*.

[35] Zhou J. J., Li D. P., Chen S. R., Luo Y., Pan H. L. (2018). The *α*2*δ*-1–NMDA receptor coupling is essential for corticostriatal long-term potentiation and is involved in learning and memory. *Journal of Biological Chemistry*.

[36] Moffett J. R., Arun P., Ariyannur P. S., Namboodiri A. M. A. (2013). N-Acetylaspartate reductions in brain injury: impact on post-injury neuroenergetics, lipid synthesis, and protein acetylation. *Frontiers in Neuroenergetics*.

[37] Moffett J. R., Ross B., Arun P., Madhavarao C. N., Namboodiri A. M. (2007). N-Acetylaspartate in the CNS: from neurodiagnostics to neurobiology. *Progress in Neurobiology*.

[38] DeVito T. J., Drost D. J., Pavlosky W. (2005). Brain magnetic resonance spectroscopy in Tourette’s disorder. *Journal of the American Academy of Child & Adolescent Psychiatry*.

[39] Boger R. H. (2014). The pharmacodynamics of L-arginine. *Alternative Therapies in Health & Medicine*.

[40] Calabrese V., Mancuso C., Calvani M., Rizzarelli E., Butterfield D. A., Giuffrida Stella A. M. (2007). Nitric oxide in the central nervous system: neuroprotection versus neurotoxicity. *Nature Reviews Neuroscience*.

[41] Shinde U. A., Mehta A. A., Goyal R. K. (2000). Nitric oxide: a molecule of the millennium. *Indian Journal of Experimental Biology*.

[42] Bradley S. A., Steinert J. R. (2016). Nitric oxide-mediated posttranslational modifications: impacts at the synapse. *Oxidative Medicine and Cellular Longevity*.

[43] Hardingham N., Dachtler J., Fox K. (2013). The role of nitric oxide in pre-synaptic plasticity and homeostasis. *Frontiers in Cellular Neuroscience*.

[44] Yun H. Y., Dawson V. L., Dawson T. M. (1997). Nitric oxide in health and disease of the nervous system. *Molecular Psychiatry*.

[45] Avcil S., Uysal P., Yenisey C., Abas B. I. (2019). Elevated melatonin levels in children with attention deficit hyperactivity disorder: relationship to oxidative and nitrosative stress. *Journal of Attention Disorders*.

[46] Gawali N. B., Chowdhury A. A., Kothavade P. S., Bulani V. D., Nagmoti D. M., Juvekar A. R. (2016). Involvement of nitric oxide in anticompulsive-like effect of agmatine on marble-burying behaviour in mice. *European Journal of Pharmacology*.

[47] Ghasemi M. (2019). Nitric oxide: antidepressant mechanisms and inflammation. *Advances in Pharmacology*.

[48] Knott A. B., Bossy-Wetzel E. (2009). Nitric oxide in health and disease of the nervous system. *Antioxidants and Redox Signaling*.

[49] Yui K., Kawasaki Y., Yamada H., Ogawa S. (2016). Oxidative stress and nitric oxide in autism spectrum disorder and other neuropsychiatric disorders. *CNS & Neurological Disorders - Drug Targets*.

[50] Fujita T., Hada T., Higashino K. (1999). Origin of D- and L-pipecolic acid in human physiological fluids: a study of the catabolic mechanism to pipecolic acid using the lysine loading test. *Clinica Chimica Acta*.

[51] Rashed M. S., Al-Ahaidib L. Y., Aboul-Enein H. Y., Al-Amoudi M., Jacob M. (2001). Determination of L-pipecolic acid in plasma using chiral liquid chromatography-electrospray tandem mass spectrometry. *Clinical Chemistry*.

[52] Beitz A. J., Larson A. A. (1985). Inhibition of intrathecally administered picrotoxin- and bicuculline-induced convulsions in mice by pipecolic acid or GABA. *European Journal of Pharmacology*.

[53] Gutirrez M. C., Delgado-Coello B. A. (1989). Influence of pipecolic acid on the release and uptake of [3H]GABA from brain slices of mouse cerebral cortex. *Neurochemical Research*.

[54] Xi L., Zhou F., Sha H. (2020). Potential plasma metabolic biomarkers of tourette syndrome discovery based on integrated non-targeted and targeted metabolomics screening. *Discovery*.

