# Supplementary material for: Potential Plasma Metabolic Biomarkers of Tourette Syndrome Discovery Based on Integrated Non-Targeted and Targeted Metabolomics Screening
Source: Evid Based Complement Alternat Med. 2022 Nov 30;2022:5080282. doi: 10.1155/2022/5080282 (PMC9894715; doi:10.1155/2022/5080282)

**Supplementary Table S1** The stand curve formulas of metabolite standards.

| component | stand curve | R2 |
| --- | --- | --- |
| L(-)-Carnitine | y=4.3390*x-69.8655 | 0.9972 |
| L-Glutamic acid hydrochloride | y=0.3286*x-1.9465 | 0.9983 |
| L-Ornithine monohydrochloride | y=1.2999*x-26.4997 | 0.9970 |
| D-Proline | y=1.0752*x+22.1328 | 0.9998 |
| L-Arginine | y=6.0785*x+156.3409 | 0.9982 |
| D-Ornithine monohydrochloride | y=1.1636*x-22.2691 | 0.9976 |
| D-Homoproline | y=5.1628*x-95.0264 | 0.9997 |
| L-Pipecolicacid | y=5.7487*x-180.3461 | 0.9975 |

**Supplementary Table S2** The AUCs of ROC through stratified 5 fold validation.

|  | Stratified sampling | D-Ornithine | D-Proline | D-Homoproline | L-Glutamate | L-Arginine | L-Ornithine | D-Pipecolinic acid | L-Carnitine |
| --- | --- | --- | --- | --- | --- | --- | --- | --- | --- |
| Sampling batch 1 | Training (60% samples) | 0.5185 | 0.5694 | 0.551 | 0.5222 | 0.6667 | 0.5291 | 0.7725 | 0.5503 |
| Sampling batch 1 | Validation (40% samples) | 0.6286 | 0.9167 | 0.7292 | 0.58 | 0.6857 | 0.6571 | 0.8769 | 0.5714 |
| Sampling batch 2 | Training (60% samples) | 0.5238 | 0.7667 | 0.797 | 0.5309 | 0.7989 | 0.5608 | 0.8556 | 0.582 |
| Sampling batch 2 | Validation (40% samples) | 0.6286 | 0.5 | 0.7142 | 0.5667 | 0.4 | 0.5429 | 0.7143 | 0.6714 |
| Sampling batch 3 | Training (60% samples) | 0.5238 | 0.7607 | 0.6391 | 0.5062 | 0.6607 | 0.5714 | 0.8167 | 0.4497 |
| Sampling batch 3 | Validation (40% samples) | 0.5 | 0.6061 | 0.4464 | 0.6667 | 0.8 | 0.5571 | 0.8143 | 0.6286 |
| Sampling batch 4 | Training (60% samples) | 0.7037 | 0.5982 | 0.5987 | 0.5309 | 0.7037 | 0.5079 | 0.7389 | 0.5926 |
| Sampling batch 4 | Validation (40% samples) | 0.6714 | 0.825 | 0.6667 | 0.6833 | 0.7143 | 0.5429 | 0.9 | 0.7714 |
| Sampling batch 5 | Training (60% samples) | 0.7714 | 0.6071 | 0.5987 | 0.5439 | 0.5661 | 0.6455 | 0.7111 | 0.4286 |
| Sampling batch 5 | Validation (40% samples) | 0.7286 | 0.925 | 0.619 | 0.7273 | 0.8571 | 0.6286 | 0.9143 | 0.6429 |

**Supplementary Figure S1**. The workflow diagram of this experiment, including preliminary screening of differential metabolites in the non-targeted group and validation in the targeted group.


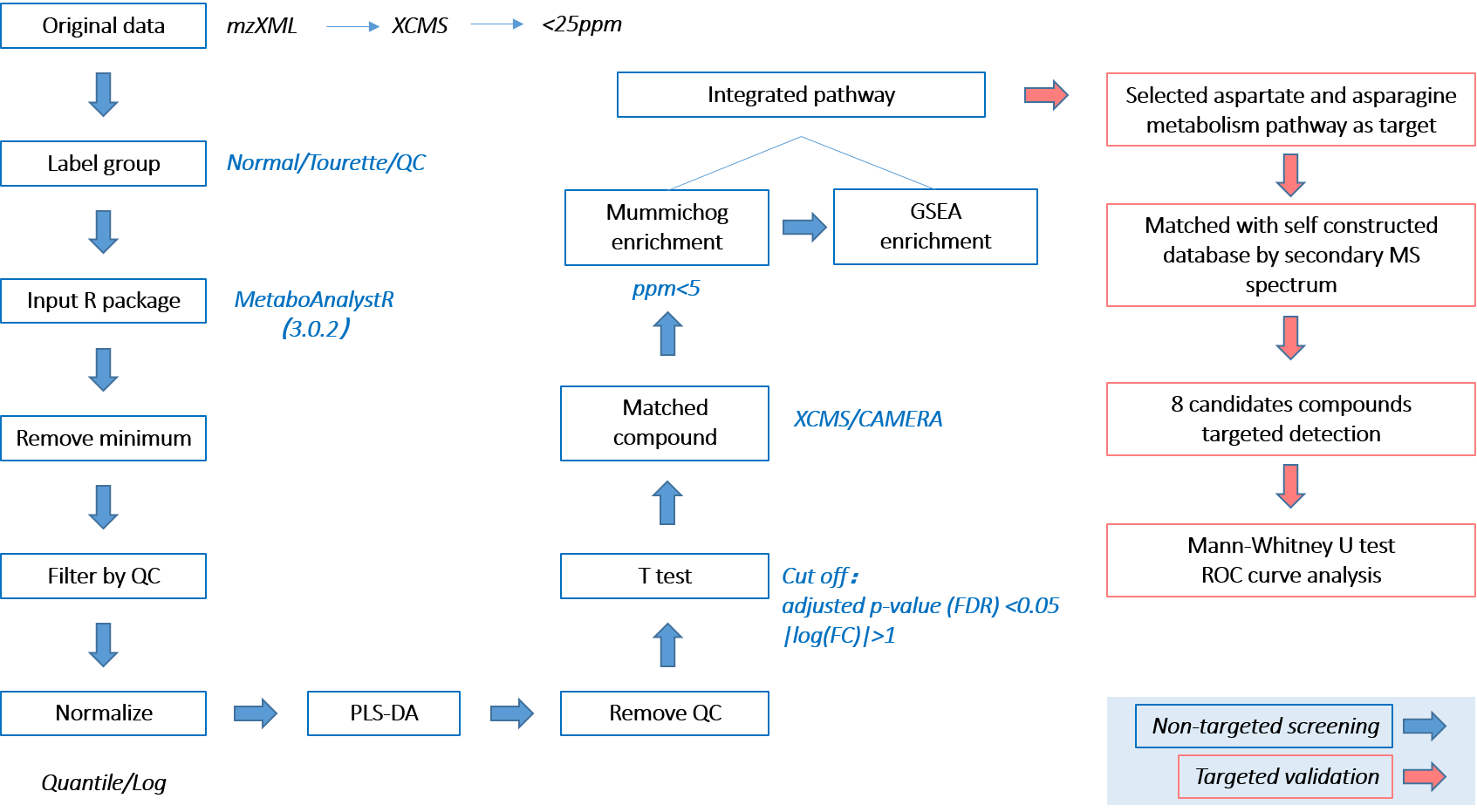


**Supplementary Figure S2**. The quantitative detection of D-Ornithine (A), D-Prolile (B), D-Homoproline (C), L-Glutamate (D), L-Arginine (E), L-Ornithine (F), D-Pipecolinic (G), L-Carnitine (H) by ESI in positive mode using MRM.


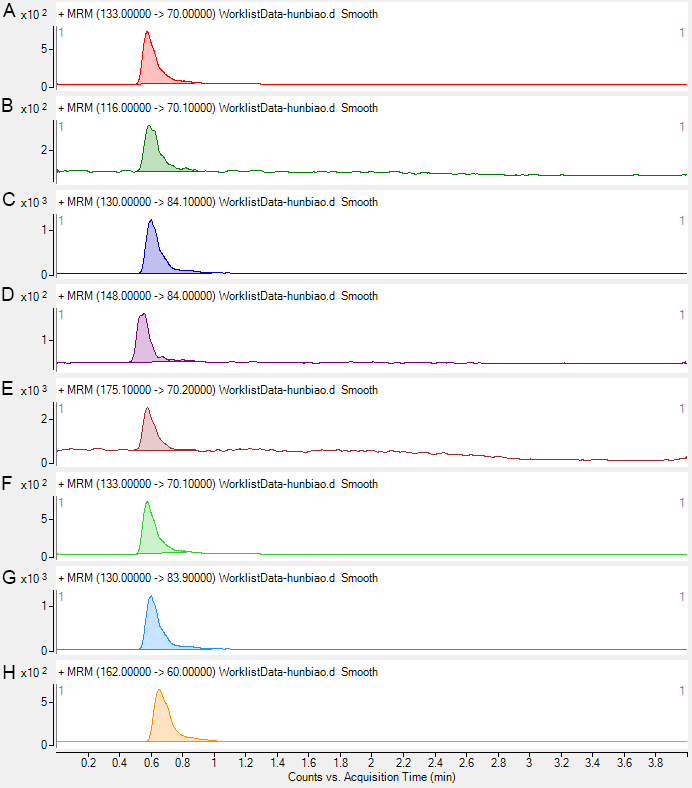


**Supplementary Figure S3.** The stand curve of metabolite standards based on UHPLC/MS/MS platform, including D-Ornithine (A), D-Prolile (B), D-Homoproline (C), L-Glutamate (D), L-Arginine (E), L-Ornithine (F), D-Pipecolinic (G), L-Carnitine (H).


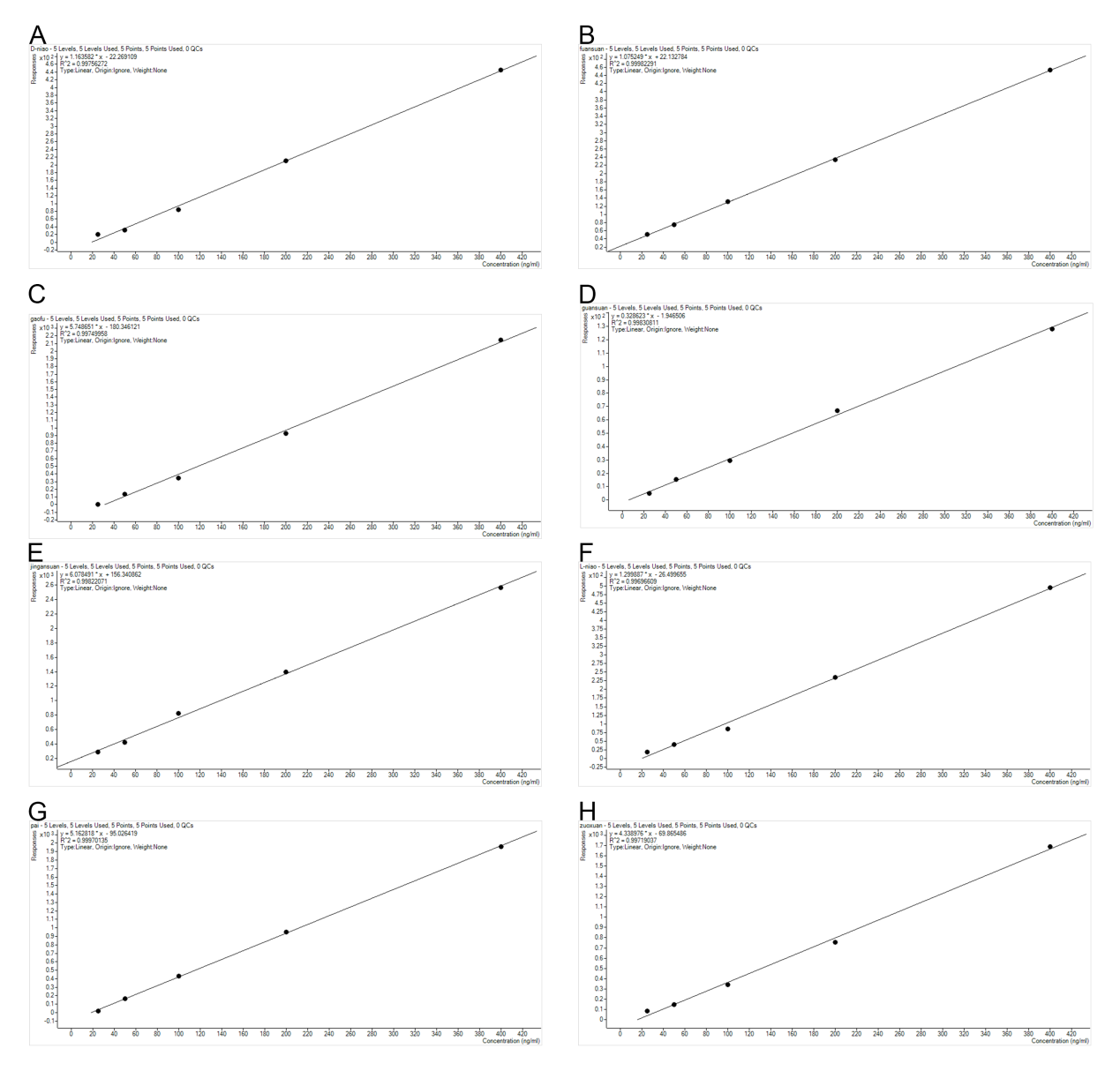

Supplement: Supplementary Materials — Supplementary Table S1: the standard curve formulas of metabolite standards. Supplementary Table S2: the AUCs of ROC through stratified 5-fold validation. Supplementary Figure S1: the workflow diagram of this experiment, including preliminary screening of differential metabolites in the nontargeted group and validation in the targeted group. Supplementary Figure S2: the quantitative detection of D-ornithine (A), D-proline (B), D-homoproline (C), L-glutamate (D), L-arginine (E), L-ornithine (F), D-pipecolinic (G), and L-carnitine (H) by ESI in the positive mode using MRM. Supplementary Figure S3: The standard curve of metabolite standards based on the UHPLC/MS/MS platform, including D-ornithine (A), D-proline (B), D-homoproline (C), L-glutamate (D), L-arginine (E), L-ornithine (F), D-pipecolinic (G), and L-carnitine (H). [file 5080282.f1.docx]
